# Baseline levels of serum high sensitivity C reactive protein and lipids in predicting the residual risk of cardiovascular events in Chinese population with stable coronary artery disease: a prospective cohort study

**DOI:** 10.1186/s12944-018-0923-1

**Published:** 2018-12-03

**Authors:** Wen Dai, Ziyu Zhang, Shuiping Zhao

**Affiliations:** Department of Cardiology, The Second Xiangya Hospital, Central South University, No. 139, Middle Renmin Road, Changsha, 410011 China

**Keywords:** Coronary artery disease, Inflammation, High sensitivity-C reactive protein, Triglyceride, High density lipoprotein cholesterol

## Abstract

**Background:**

The contributions of inflammation, triglyceride (TG) and high-density lipoprotein cholesterol (HDL-C) to the residual risk of cardiovascular events have not been determined in a large cohort of Chinese population before. This study was aimed to investigate the association of serum levels of high sensitive C reactive protein (hs-CRP), TG and HDL-C with the residual risk of cardiovascular events in patients with stable coronary artery disease (CAD).

**Methods:**

We enrolled 4090 patients with stable CAD from 13 hospitals in China. All participants received optimal medical treatment (OMT) for stable CAD suggested by guidelines and were followed. The endpoint measures were the first occurrence of a major adverse cardiovascular event (MACE), defined as cardiovascular death, non-fatal myocardial infarction, non-fatal stroke or unplanned coronary revascularization. Cox proportional regression analysis was conducted to identify independent predictors of MACE.

**Results:**

We found that hs-CRP and HDL-C levels were associated with coronary lesion severity at baseline (both *p* < 0.001). After 3 months OMT, 91.2% (3730/4090) patients achieved the therapeutic goal for low density lipoprotein cholesterol (LDL-C) (< 1.8 mmoL/L). During a mean follow-up period of 39.5 months, 11.5% (471/4090) patients suffered MACE. In multivariate Cox proportional regression analysis, the hazard ratio for MACE was 1.17 (95% confidence interval: 1.07–1.28, *p* < 0.001) per standardized deviation in the log-transformed hs-CRP levels after adjustment for other traditional cardiovascular risk factors. However, baseline TG and HDL-C levels were not associated with MACE in this study.

**Conclusions:**

Baseline hs-CRP level was an independent predictor of residual risk of cardiovascular events in Chinese population with stable CAD. However, TG and HDL-C levels were not associated with MACE.

## Background

Low density lipoprotein cholesterol (LDL-C) is a well-established pathogenic risk factor of coronary artery disease (CAD) [1, 2]. However, residual cardiovascular risk in population is still remained in the setting of controlled plasma LDL-C level and thus has been a major concern [3, 4]. Therefore, efforts to identify other modifiable risk factors to further reduce residual cardiovascular risk are needed.

Inflammation has long been regarded as a critical participant in CAD development. More importantly, the recent trial has demonstrated that treatment with canakinumab targeting interleukin-1β (IL-β), an important component of immune reaction, significantly reduced cardiovascular event rate [5]. Thus, anti-inflammation therapy emerges as a novel intriguing approach to deal with CAD in clinical practice.

The role of high-density lipoprotein cholesterol (HDL-C) and triglycerides (TGs) in CAD still remain controversial. Although observational studies found that HDL-C and TG levels are both closely associated with the risk of CAD [6, 7], randomized controlled trials have failed to show that medications designed to increase HDL-C or decrease TG levels have any significant clinical benefits [8–13]. Interestingly, several large-scale genetic studies have consistently shown that some variants in gene loci involved in serum TG metabolism are associated with the risk of CAD [14–16], leading to the renewed interests of researchers on unraveling the role of TG in CAD.

However, the contribution of inflammation, TG and HDL-C to the residual risk of cardiovascular events have not been determined in a large cohort of Chinese population before. Cardiovascular risk attributed to inflammation and those serum lipid parameters may vary among different ethnics due to the differences in genetic background and life style.

Therefore, we investigated the values of serum high sensitivity C reactive protein (hs-CRP), a routine inflammatory biomarker for cardiovascular risk assessment, TG and HDL-C in predicting major adverse cardiovascular event (MACE) in a cohort of Chinese population with stable CAD who received optimal medical treatment (OMT).

## Methods

### Study design and population

**Fig. 1:**
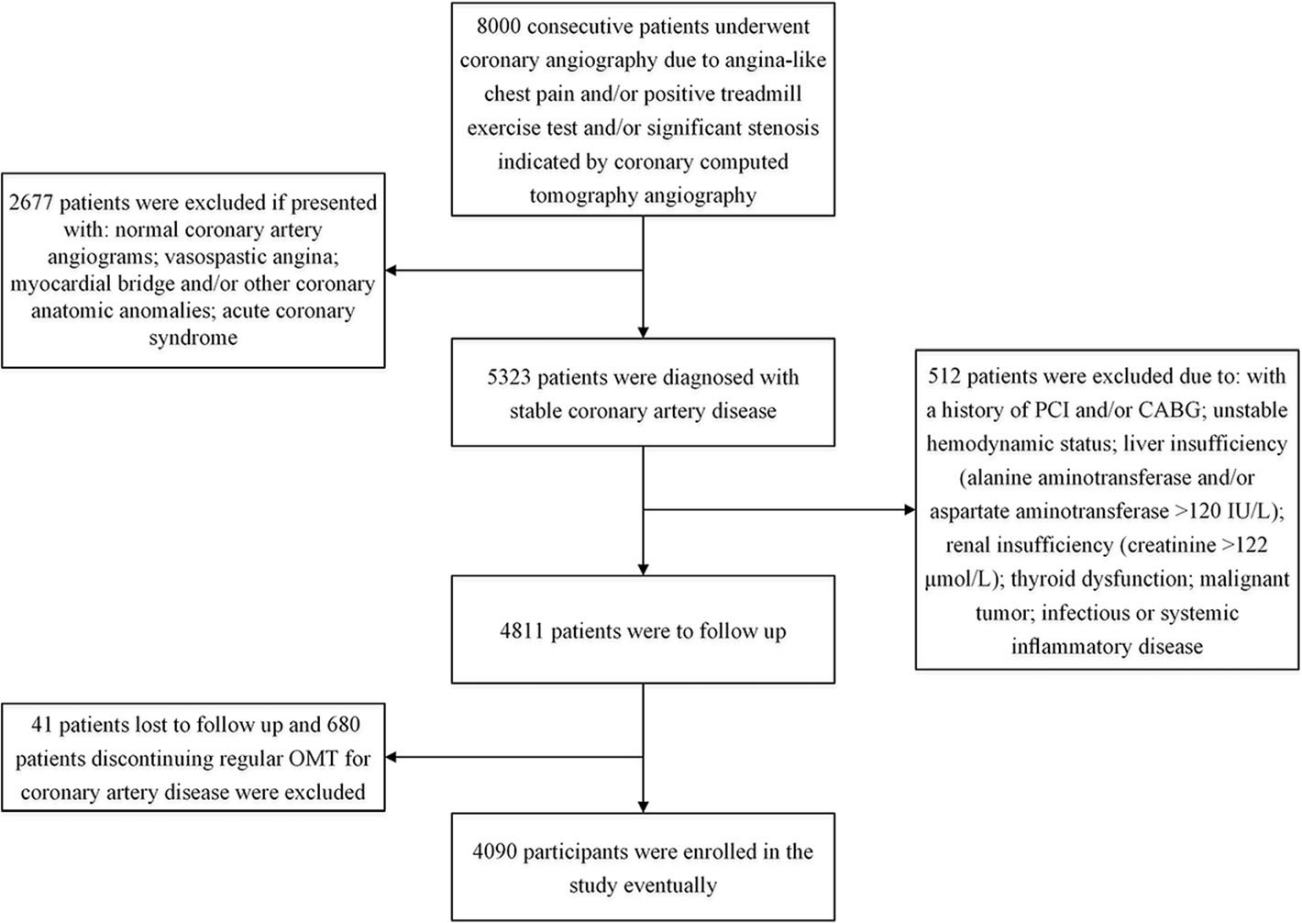
The selection process of study population

This is a prospective cohort study. Initially, we recruited patients with stable CAD from 13 hospitals in China from February 2013 to December 2013. Their baseline characteristics were recorded. Afterwards, all participants were followed by study investigators and the data of MACE were collected. Statistical analyses were conducted to investigate the association between baseline levels of hs-CRP, TG and HDL-C and MACE. The selection process of study population was illustrated in Fig. 1.

As described in previous study [17], patients were diagnosed with stable CAD if they presented with one of the following clinical phenotypes as a result of significant coronary artery atherosclerotic stenosis: 1) stable angina: chest pain precipitated by physical activity that remits with rest; 2) ischemic cardiomyopathy: cardiomyopathy caused by the atherosclerotic narrowing of coronary arteries; 3) latent coronary artery disease: disease characterized by myocardial ischemia and coronary stenosis that are identifiable by medical tests but not with apparent clinical symptoms [18, 19]. Contrarily, acute coronary syndrome (including unstable angina and myocardial infarction), vasospastic angina, and microvascular angina were not considered into the scope of stable CAD. Patients were excluded from the study for the criteria as follows: with myocardial bridge and/or other coronary anatomic anomalies; history of percutaneous coronary intervention (PCI) and/or coronary artery bypass grafting (CABG); unstable hemodynamic status; renal insufficiency (creatinine > 122 μmol/L); liver insufficiency (alanine aminotransferase and/or aspartate aminotransferase > 120 IU/L); thyroid dysfunction; infectious or systemic inflammatory disease; malignant tumor; discontinue regular OMT for stable CAD during follow-up; lost to follow-up.

This study was approved by the ethics committee review board of The Second Xiangya Hospital of Central South University. The study was carried out in accordance with the 1975 Declaration of Helsinki and the relevant regulations. Informed written consent was obtained from all participants.

### Data collection

Demographic characteristics, serum levels of lipids and hs-CRP, and coronary atherosclerosis severity at baseline were recorded. Data of MACE of participants were collected during follow-up period. We also measured the serum levels of lipids and hs-CRP at the end of the 12-week after recruitment. The following events were considered MACE: 1) cardiovascular deaths: deaths attributable to cardiovascular causes; 2) non-fatal myocardial infarctions: myocardial infarctions that did not result in death; 3) non-fatal strokes: strokes that did not result in death; and 4) unplanned coronary revascularizations: unscheduled PCI or CABG. The study investigators obtained follow-up information at regular intervals via face-to-face or telephone interviews. The follow-up period lasted from the time of recruitment to January 2017 or the date of a MACE.

All participants were prescribed with OMT for stable CAD, suggested by recent guidelines [1], during the follow-up period as follows: 1) antithrombotic agents: aspirin and/or clopidogrel; 2) anti-ischemic agents: nitrates and/or beta-receptor blockers (β-blockers) and/or calcium channel-blocking (CCB) agents; 3) renin-angiotensin inhibitors; and 4) LDL-C-lowering agents: statins.

As described in our previous study [17], blood samples were drawn by venipuncture after at least 10 h of overnight fasting. The blood specimens were processed and assessed at the central laboratory in each hospital. All clinical laboratories included in this study were standardized and certified. An automatic biochemistry analyzer (Hitachi 7360; Hitachi Ltd., Tokyo, Japan) and commercially available agents were used to measure serum total cholesterol (TC), LDL-C, HDL-C, TG, hs-CRP, fasting glucose and Hemoglobin A1c (HbA1c) levels. Hs-CRP and HbA1c levels were measured via turbidimetric immunoassay, and TC, LDL-C, HDL-C, TG, and glucose levels were measured using enzymatic assay. The left ventricular ejection fraction (LVEF) was determined by cardiac ultrasound examination. Coronary angiographic data were collected from patient catheterization laboratory records by at least 3 interventional cardiologists. Coronary lesion severity was assessed in each patient by the Gensini score (GS) [20], which was calculated by scoring each atherosclerotic lesion according to the degree of coronary artery luminal narrowing and the location of the lesion. The total score was calculated as a sum of the product of the stenosis and location score of each affected lesion.

The traditional risk factors for CAD were defined as described in our previous studies [17, 21]. Hypertension was defined as blood pressure ≥ 140/90 mmHg in more than two measurements and/or the requirement of treatment with anti-hypertension drugs. Diabetes mellitus was defined as fasting serum glucose levels ≥7.0 mmol/L, and/or random serum glucose ≥11.1 mmol/L, and/or 2-h post-prandial serum glucose ≥11.1 mmol/L on the oral glucose tolerance test in multiple determination and/or the requirement of treatment with hypoglycemic agents. The BMI was calculated as weight divided by height squared. Current smokers were subjects who had smoked regularly within the previous 12 months.

All study investigators underwent a training program and fully understood the aims of the study and the processes and methodologies used to collect the data.

### Statistical analysis

Numerical variables were expressed as the mean ± standard deviation (SD) or as medians (Q1-Q3 quartiles), according to the data distribution. Categorical variables were expressed as numbers (percentage). Differences in numerical variables between groups were analyzed by the independent *t* test, analysis of variance (ANOVA), the Mann-Whitney *U* test or the Kruskal-Wallis *H* test, as appropriate, and differences in categorical variables were analyzed by the chi-square test. Multiple linear regression was used to estimate the associations between coronary lesion severity and levels of serum lipid parameters and hs-CRP. Cox proportional hazard regression analysis was performed to examine the association between MACE and levels of serum lipid parameters and hs-CRP. TG and hs-CRP levels were log-transformed in the multiple linear regression and Cox regression analysis due to the positively skewed nature of the distribution. SPSS software (version 20.0; SPSS Inc., Cary, Chicago, USA) was used to perform the statistical analyses. For all analyses, two-tailed *p* values < 0.05 were considered statistically significant.

## Results

### Baseline characteristics

A total of 4090 patients with stable CAD were enrolled in this study. As shown in Table 1, they were categorized into tertile subgroups according to their GS. There were significant differences in the levels of TC, LDL-C, HDL-C, TG and hs-CRP among the three subgroups (all *p* < 0.01). The subgroups also differed significantly with respect to age, the percentage of male patients, systolic pressure, smoking status, hypertension and diabetes history, and fasting glucose and HbA1c levels (all *p* < 0.01).

**Table 1:** Baseline characteristics of the study population according to Gensini score tertiles

	Overall (*n* = 4090)	Gensini score category			*p*
		< 26	26–43	≥44	
		(*n* = 1309)	(*n* = 1405)	(*n* = 1376)	
Clinical characteristics					
Age, years	61.1 ± 10.9	58.9 ± 10.8	61.8 ± 10.7	62.6 ± 10.8	< 0.001
Gender (male), % (n)	67.9(2779)	62.9 (823)	68.4 (961)	72.3 (995)	< 0.001
BMI, kg/m^2^	25.0 ± 3.2	25.0 ± 3.3	25.0 ± 3.1	25.1 ± 3.1	0.262
Systolic pressure, mm Hg	134 ± 20	132 ± 19	134 ± 20	135 ± 21	0.002
Diastolic pressure, mm Hg	78 ± 12	78 ± 12	78 ± 12	78 ± 13	0.726
Hypertension, % (n)	57.8(2362)	52.6 (689)	58.9 (827)	61.5 (846)	< 0.001
Diabetes mellitus, % (n)	23.9(977)	22.2 (290)	22.4 (315)	27.0 (372)	0.003
Current smoking, % (n)	35.2(1438)	31.0 (406)	34.2 (480)	40.1 (552)	< 0.001
Family history of premature CAD, % (n)	145 (3.5)	45 (3.4)	60 (4.3)	40 (2.9)	0.146
LVEF, %	61.1 ± 6.9	61.3 ± 7.0	61.0 ± 6.9	61.0 ± 6.8	0.594
Biochemistry parameters					
TC, mmol/L	4.64 ± 1.08	4.56 ± 1.02	4.61 ± 1.05	4.74 ± 1.16	< 0.001
LDL-C, mmol/L	2.78 ± 0.91	2.68 ± 0.84	2.74 ± 0.90	2.91 ± 0.96	< 0.001
HDL-C, mmol/L	1.14 ± 0.31	1.18 ± 0.32	1.12 ± 0.31	1.12 ± 0.30	< 0.001
TG, mmol/L	1.51 (1.07–2.18)	1.48 (1.06–2.12)	1.46 (1.05–2.10)	1.57 (1.12–2.28)	0.003
Hs-CRP, mg/L	2.63 (1.10–7.10)	2.47 (1.00–6.37)	2.41 (1.06–6.66)	2.99 (1.23–8.39)	< 0.001
Fasting glucose, mmol/L	6.26 ± 2.39	6.00 ± 2.11	6.27 ± 2.37	6.50 ± 2.62	< 0.001
HbA1C, (%)	6.4 ± 1.4	6.3 ± 1.4	6.4 ± 1.4	6.6 ± 1.5	< 0.001

Data are shown as mean ± standard deviation, median (Q1–Q3 quartiles), or percentages (n). *P* values from analysis of the variance (ANOVA), Kruskal-Wallis H tests, or chi-square tests. Two-tailed *p* < 0.05 was considered statistically significant. *CAD* coronary artery disease, *BMI* body mass index, *LVEF* left ventricular ejection fraction, *TC* total cholesterol, *LDL-C* LDL cholesterol, *HDL-C* HDL cholesterol, *TG* triglyceride, *hs-CRP* high-sensitivity C reactive protein, *HbA*1*C* Hemoglobin A1c

### Coronary lesion severity

We evaluated the association between GS and metabolic risk factors using multiple linear regression analysis (Table 2). We found that LDL-C and hs-CRP were positively associated with GS (both *p* < 0.001). By contrast, HDL-C was negatively associated with GS (p < 0.001). However, there were no associations between GS and levels of TC and TG in this study. Age, male gender, hypertension, diabetes and current smoking history were all positively associated with the GS (all *p* < 0.05) (Table 2).

**Table 2:** Multiple linear regression analysis for the association of metabolic risk factors with coronary severity

Variables	Standardized coefficients	*p*
Age	0.188	< 0.001
Gender (male vs. female)	0.082	< 0.001
BMI	−0.001	0.963
History of hypertension (with vs. without)	0.033	0.031
History of diabetes mellitus (with vs. without)	0.048	0.002
History of current smoking (with vs. without)	0.088	< 0.001
Family history of premature CAD	−0.009	0.537
TC	−0.045	0.267
LDL-C	0.161	< 0.001
HDL-C	−0.077	< 0.001
TG (log-transformed)	0.028	0.185
Hs-CRP (log-transformed)	0.166	< 0.001

*P* values were from linear regression. Two-tailed *p* < 0.05 was considered statistically significant. *CAD* coronary artery disease, *BMI* body mass index, *TC* total cholesterol, *LDL-C* LDL cholesterol, *HDL-C* HDL cholesterol, *TG* triglyceride, *hs-CRP* high-sensitivity C reactive protein

### Levels of serum lipids and hs-CRP after 12-week OMT

We measured the levels of lipid parameters and hs-CRP of participants after 12-week OMT. There were significant decreases in TC, LDL-C, TG and hs-CRP levels, and increases in HDL-C levels (All p < 0.05). It should be noted that 91.2% (3730/4090) patients achieved the therapeutic goal for LDL-C (< 1.8 mmoL/L).

### Mace

We found that 471 (11.5%) patients experienced MACE within a mean follow-up period of 39.5 months. Among them, 56 (1.4%) patients suffered cardiovascular deaths, 138 (3.4%) patients suffered non-fatal myocardial infarctions, 34 (0.8%) patients suffered non-fatal strokes, and 243 (5.9%) patients underwent unplanned coronary revascularization.

**Fig. 2:**
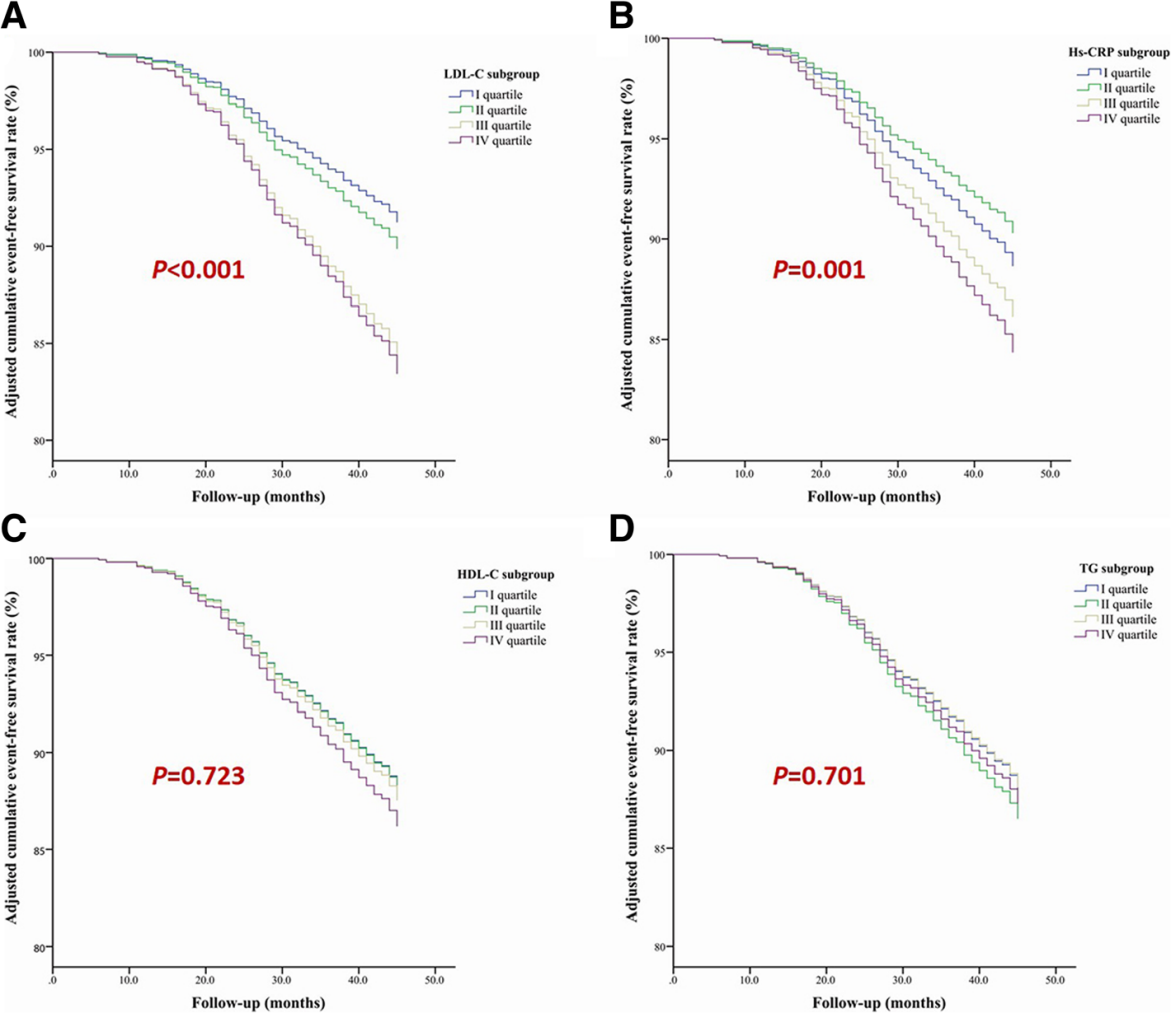
The adjusted cumulative event-free survival rate of the study population. There existed significant differences in the adjusted cumulative event-free survival rate among LDL-C and hs-CRP subgroups (*p* = 0.001).LDL-C: low density lipoprotein cholesterol, hs-CRP: high sensitivity C reactive protein; HDL-C: high density lipoprotein cholesterol; TG: triglyceride

To determine whether baseline levels of hs-CRP, HDL-C and TG were associated with MACE, we performed Cox proportional hazard regression analysis. In the univariate analysis (Table 3), hs-CRP was associated with MACE, while HDL-C and TG were not. Furthermore, in the multivariate analysis adjusting for traditional risk factors, we found that there was a significant difference in the adjusted event-free survival rate among the hs-CRP quartile subgroups (*p* = 0.001) (Fig. 2). The hazard ratio for MACE was 1.17 (95% confidence interval: 1.07–1.28, *p* < 0.001) per 3.9-fold increase in the hs-CRP concentration [i.e., per one standardized deviation increase in the log-transformed hs-CRP level] (Table 4). The LDL-C was also associated with MACE (p < 0.001). However, HDL-C and TG levels were not predictors of MACE. We also noticed that diabetes was positively and coronary revascularization was negatively associated with MACE (both *p* < 0.05).

**Table 3:** Univariate Cox regression analysis for the predictors of major adverse cardiovascular event

Variables	HR	95% CI	*p*
Age	0.99	0.99–1.00	0.117
Gender (male vs. female)	1.05	0.87–1.28	0.596
BMI	1.02	0.99–1.05	0.141
History of hypertension (with vs. without)	1.10	0.91–1.32	0.321
History of diabetes mellitus (with vs. without)	1.64	1.35–1.99	< 0.001
History of current smoking (with vs. without)	1.15	0.95–1.38	0.153
Family history of premature CAD	1.22	0.78–1.92	0.376
Coronary revascularization	0.82	0.68–0.98	0.028
LVEF, %	1.00	0.99–1.01	0.851
TC	1.25	1.16–1.35	< 0.001
LDL-C	1.37	1.27–1.48	< 0.001
HDL-C	1.15	0.86–1.53	0.344
TG (log-transformed)	1.07	0.92–1.25	0.363
Hs-CRP (log-transformed)	1.19	1.09–1.30	< 0.001

*P* values were from Cox proportional hazard regression. Two-tailed *p* < 0.05 was considered statistically significant. *CAD* Coronary artery disease, *BMI* body mass index, *TC* total cholesterol, *LDL-C* LDL cholesterol, *HDL-C* HDL cholesterol, *TG* triglyceride, *hs-CRP* high-sensitivity C reactive protein, *HR* hazard ratio, *CI* confidence interval

**Table 4:** Multivariate Cox regression analysis for the independent predictors of major adverse cardiovascular event

Variables	HR	95% CI	*p*
Age	1.00	0.99–1.01	0.600
Gender (male vs. female)	1.09	0.86–1.36	0.483
BMI	1.00	0.97–1.03	0.754
History of hypertension (with vs. without)	1.09	0.90–1.32	0.366
History of diabetes mellitus (with vs. without)	1.69	1.39–2.05	< 0.001
History of current smoking (with vs. without)	1.06	0.85–1.32	0.595
Family history of premature CAD	1.20	0.77–1.89	0.420
Coronary revascularization	0.82	0.68–0.98	0.027
LVEF, %	1.00	0.99–1.01	0.770
TC^a^	0.78	0.60–1.01	0.056
LDL-C^a^	1.63	1.30–2.05	< 0.001
HDL-C^a^	1.10	0.97–1.24	0.125
TG^a^ (log-transformed)	1.06	0.93–1.21	0.375
Hs-CRP ^a^ (log-transformed)	1.17	1.07–1.28	< 0.001

*P* values were from Cox proportional hazard regression. Two-tailed *p* < 0.05 was considered statistically significant. *CAD* Coronary artery disease, *BMI* body mass index, *TC* total cholesterol, *LDL-C* LDL cholesterol, *HDL-C* HDL cholesterol, *TG* triglyceride, *hs-CRP* high-sensitivity C reactive protein, *HR* hazard ratio, *CI* confidence interval. ^a^ One standard deviation increase

## Discussion

In this prospective study, we found that, baseline hs-CRP level was an independent predictor of MACE in a cohort of Chinese population with stable CAD who received OMT within a mean follow-up period of 39.5 months. However, HDL-C and TG levels were not associated with MACE. Our findings can serve as new evidences for the contribution of inflammation to residual cardiovascular risk in Chinese population.

Residual cardiovascular risk in the setting of adequately controlled LDL-C has been a major concern for the treatment of CAD. In this study, 11.5% (471/4090) of patients, suffered MACE despite receiving OMT during an average of 39.5 months fellow-up period. It should be noted that 91.2% (3730/4090) patients had achieved the therapeutic goal for LDL-C (< 1.8 mmol/L) after 3 month OMT. The finding was similar to those of our previous studies in Chinese population [22, 23].

LDL-C is a well- established risk factor of CAD. It has been shown that lowering of circulated LDL-C dose dependently results in reduction of cardiovascular events. Every 1 mmol/L reduction in LDL-C is associated with a corresponding 22% decrease in CAD mortality and morbidity [2]. Consistently, we found that baseline LDL-C was associated with MACE in this study.

Substantial experimental work has elucidated molecular and cellular pathways of inflammation that promote atherosclerosis [24]. Evidences from epidemiologic studies indicated that several inflammatory makers, including hs-CRP, interleukin-6 (IL-6), tumor necrosis factor-α (TNF-α), etc., were positively associated with cardiovascular risk in western population [25–27]. Also, patients with insulin resistance, regarded as a chronic inflammatory event, had increased risk of cardiovascular events [28, 29]. Contrarily, some molecules that are known to have anti-inflammatory action, e.g. Ghrelin, have shown some beneficial effects on cardiovascular system [30]. Of which, hs-CRP is the most well-recognized and widely-used inflammatory marker for cardiovascular risk assessment. Paul et al. found that, of the 12 markers measured, hs-CRP was the strongest univariate predictor of the risk of cardiovascular events [25]. More importantly, it has been recently reported that pharmacological inhibition of inflammation with canakinumab reduced cardiovascular events in CAD patients with hs-CRP levels of more than 2 mg/L [5]. Thus, the newly released clinical practice guideline from American College of Cardiology/American Heart Association recommends elevated hs-CRP level (≥2 mg/L) as a risk-enhancing factor when assessing cardiovascular risk [31]. Our study indicated that hs-CRP level was an independent predictor of residual risk of cardiovascular events in Chinese population. Future clinical trials can testify the benefits of anti-inflammation therapy in Chinese patients with CAD.

Nevertheless, we found that the baseline levels of TG and HDL-C were not associated with MACE. Elevated TG and decreased HDL-C are more frequently seen than high LDL-C level in Chinese population [32]. The role of TG and HDL-C in CAD still remain controversial. Although observational studies have suggested serum TG level is associated with CAD risk, randomized controlled trials of fibrates and omega-3 fatty acids to reduce TG failed to show any significant clinical benefits [9, 11]. The fact that atherosclerotic plaques possess primarily cholesterol instead of TG also objects to the premise that TG is directly involved in plaque formation. However, recent genetic studies showed some variants in gene loci involved in plasma TG metabolism are associated with the risk of CAD [14–16]. Genetic evidence is free of confounders and reverse causation and is thus helpful for identifying causal risk factors for CAD. Therefore, renewed interests are gained on these gene loci and corresponding encoding products, such as apolipoprotein C3 [15, 16]. Future research at this point will provide new insight to the understanding of the role of TG in CAD. HDL exerts various athero-protective properties, including mediating cholesterol efflux, protecting vascular endothelium, anti-inflammatory and anti-apoptotic effects [7, 33]. However, medication aimed to increase HDL-C levels didn’t reduce CAD risk [8, 10, 12, 13]. Genetic studies also didn’t support HDL-C as a risk factor of CAD [7]. Recent studies suggested that it is HDL function but not HDL-C levels that play a role in CAD development [7].

Due to the observational nature of this study, we could not determine the benefits of treatment to reduce hs-CRP or TG, or increase HDL-C levels in Chinese population. Future clinical trials will provide more comprehensive evidences.

## Conclusions

In summary, our study demonstrated that, baseline hs-CRP levels was an independent risk factor for MACE in a cohort of Chinese population with stable CAD who received OMT. However, TG and HDL-C levels were not associated with MACE.

## Abbreviations

ANOVA: analysis of variance
CABG: coronary artery bypass grafting
CAD: coronary artery disease
CCB: calcium channel-blocking
GS: Gensini score
HbA1c: Hemoglobin A1c
HDL-C: high-density lipoprotein cholesterol
hs-CRP: high sensitivity C reactive protein
IL-6: interleukin-6
IL-β: interleukin-1β
LDL-C: low density lipoprotein cholesterol
LVEF: left ventricular ejection fraction
MACE: major adverse cardiovascular event;
OMT: optimal medical treatment
PCI: percutaneous coronary intervention
SD: standard deviation
TC: total cholesterol
TG: triglyceride
TNF-α: tumor necrosis factor-α
β-blockers: beta-receptor blockers
